# A Modified Version of the Transactional Stress Concept According to Lazarus and Folkman Was Confirmed in a Psychosomatic Inpatient Sample

**DOI:** 10.3389/fpsyg.2021.584333

**Published:** 2021-03-05

**Authors:** Nina Obbarius, Felix Fischer, Gregor Liegl, Alexander Obbarius, Matthias Rose

**Affiliations:** ^1^Department of Psychosomatic Medicine, Center for Internal Medicine and Dermatology, Charité – Universitätsmedizin Berlin, Berlin, Germany; ^2^Dornsife Center for Self-Report Science, University of Southern California, Los Angeles, CA, United States; ^3^Department of Quantitative Health Sciences, Outcomes Measurement Science, University of Massachusetts Medical School, Worcester, MA, United States

**Keywords:** stress, depression, Lazarus, transactional model, structural equation model

## Abstract

**Background:**

Stress is a major risk factor for the impairment of psychological well-being. The present study aimed to evaluate the empirical evidence of the Transactional Stress Model proposed by Lazarus and Folkman in patients with psychosomatic health conditions.

**Methods:**

A structural equation model was applied in two separate subsamples of inpatients from the Department of Psychosomatic Medicine (total *n* = 2,216) for consecutive model building (sample 1, *n* = 1,129) and confirmatory analyses (sample 2, *n* = 1,087) using self-reported health status information about perceived stress, personal resources, coping mechanisms, stress response, and psychological well-being.

**Results:**

The initial model was created to reflect the theoretical assumptions by Lazarus and Folkman about their transactional stress concept. This model was modified until a sufficient model fit was reached (sample 1: CFI = 0.904, TLI = 0.898, RMSEA = 0.072 [0.071–0.074], SRMR = 0.061). The modified model was confirmed in a second sample (sample 2: CFI = 0.932, TLI = 0.928, RMSEA = 0.066 [0.065–0.068], SRMR = 0.052). Perceived external stressors and personal resources explained 91% of the variance of the stress response, which was closely related to symptoms of depression (63% variance explained). The attenuating effect of resources on stress response was higher (standardized β = -0.73, *p* < 0.001) than the impact of perceived stressors on stress response (standardized β = 0.34, *p* < 0.001).

**Conclusion:**

The empirical data largely confirmed the theoretical assumption of the Transactional Stress Model, which was first presented by Lazarus and Folkman, in patients with a wide range of psychosomatic conditions. However, data analyses were solely based on self-reported health status. Thus, proposed inner psychological mechanisms such as the appraisal process could not be included in this empirical validation. The operationalization and understanding of coping processes should be further improved.

## Introduction

The impact of psychological stress on health has been widely confirmed (Glaser and Kiecolt-Glaser, 2005; de Vente et al., 2006; Bengtsson et al., 2009; Lupien et al., 2009; Hemmerle et al., 2012; Gradus, 2017; Sgoifo et al., 2017). Stress is a major risk factor for the development of mental disorders such as major depression (Kessler, 1997; Uehara et al., 1999; Mino et al., 2006; Hankin et al., 2015), representing one of the most burdensome diseases worldwide (Lim et al., 2012). Yet, the pathway that connects stress exposure to a manifest disease is not well understood. It remains largely unknown why, in the face of adversity, some people turn ill whereas others remain healthy. Given the high number of stress-associated diseases (Forsen, 1991; Grassi et al., 2002), a better understanding of underlying stress processes is urgently needed. In disease prevention, knowledge about different response patterns to stress are highly important for the early identification of people in need of medical care (Caffo et al., 2008). In addition, individualized interventions could be designed based on an improved understanding of the underlying mechanisms in stress response. Therefore, in this study, we seek to empirically confirm or reject stress pathways as suggested by a common stress model.

In the resilience literature, a huge number of protective factors were identified that enable an individual to overcome adversity without negative consequences (Caffo et al., 2008; Davydov et al., 2010; Windle, 2011; Fletcher and Sarkar, 2013). These protective factors include sense of coherence, self-efficacy, and optimism (Eriksson and Lindstrom, 2006; Hart et al., 2006; Surtees et al., 2006; Kroninger-Jungaberle and Grevenstein, 2013; Braun-Lewensohn and Sagy, 2014; Campo et al., 2017; Kim et al., 2017). Over the past years, the focus of resilience research moved away from the identification of protective factors toward the understanding of underlying resilience processes (Luthar et al., 2000). Hence, a closer look at the pathways between stressors, resources, coping, stress response, and mental health is required to better understand how resilience impacts coping with stressors.

**FIGURE 1:**
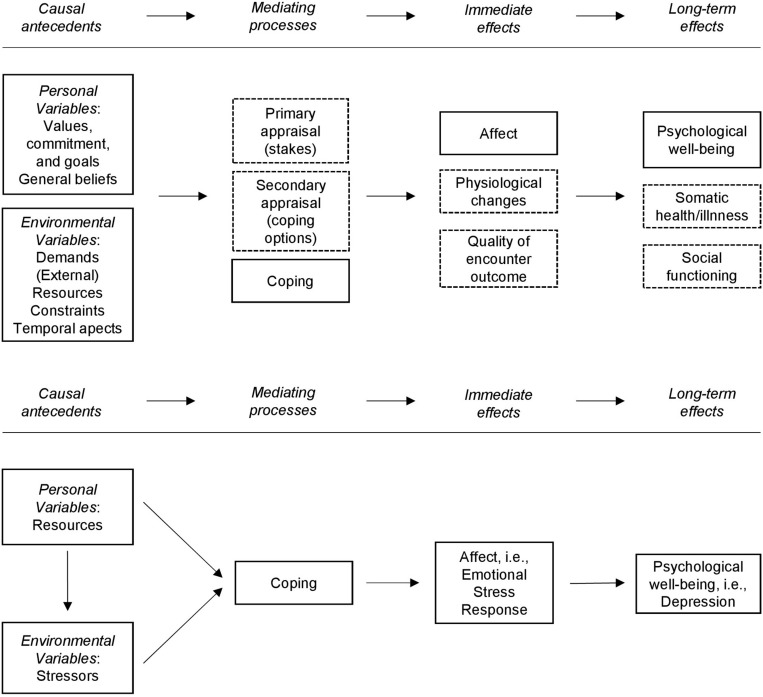
Transactional model by Lazarus and Folkman (1987). Dashed frames indicate parts of the models that were not tested in the present study. Bottom: parts of the model that were tested in the present study.

One of the most popular models describing stress pathways has been proposed by Lazarus and Folkman as early as in 1987 with the first reports dating back to 1966 (Lazarus, 1966; Lazarus and Folkman, 1984, 1987). Their Transactional Model provides the theoretical framework for the present study and is depicted in Figure 1 (upper model). The model emphasizes the person–environment transaction and suggests that a stress response is highly influenced by individual appraisal processes. Once confronted with stressors, the individual evaluates the relevance of the stressors (primary appraisal) and its own resources to overcome stress (secondary appraisal). Primary and secondary appraisals are believed to have an impact on the coping strategies chosen by the individual. Coping affects the immediate stress response as well as long-term health, psychological well-being, and social functioning. For simplicity, the authors depicted a linear section of the whole complex dynamic model and indicated the recursive nature of the model and the parallelism of the short- and long-term effects as footnotes to the figure (Lazarus and Folkman, 1987).

Later, Lazarus and his co-authors formulated a cognitive–motivational–emotional theory that refined the analysis of specific different appraisal processes leading to different emotions (Smith and Lazarus, 1990). The transactional stress concept was embedded in the larger context of emotion theory (Smith and Lazarus, 1990; Lazarus, 1993), claiming to integrate stress and emotion research. In this study, we focus on the core piece of the transactional stress theory: the person–environment transaction. Consequently, we did not analyze specific appraisal processes and emotions, but rather the pathways from personal and environmental variables via coping to stress response and mental health.

Since the authors themselves evaluated the Transactional Model and confirmed the impact of personality factors, appraisals, and coping on psychological symptoms (Folkman et al., 1986), the entire Transactional Stress Model by Lazarus and Folkman (1987) has been tested in several studies. Despite of the number of previous studies, there are several drawbacks that suggest further empirical investigation of the structure of the Transactional Stress Model. For example, a majority of the studies only assessed parts of the theoretical model (Terry, 1991, 1994; Varni and Katz, 1997; Shewchuk et al., 1999; Zureck et al., 2015; Paek et al., 2016). Also, the studies that aimed to test the whole Transactional Model differed considerably in terms of the constructs included in the model, the operationalization of those constructs and the study populations, which reduces the comparability of the results (see Supplementary Table 1 for an overview). In addition, several studies used hierarchical linear regression analyses (Quine and Pahl, 1991; Honey et al., 2003; Bouchard et al., 2004; Hulbert-Williams et al., 2013) which rendered the inclusion of complex interactions (such as mediation) within the Transactional Model impossible. The studies that applied structural equation modeling often did not use the original conceptualization of the Transactional Model. For example, Kocalevent et al. (2007) simplified the complex model by specifying only three latent factors (“Resources,” “Stress Perception,” and “Health”). In addition, the authors varied the operationalization and naming of the three latent factors throughout the studies [Heinen et al. (2017): “Personal resources,” “Perceived Stress,” “Emotional Distress”; Kocalevent et al. (2013): “Resources,” “Chronic stress,” “Fatigue”; Kocalevent et al. (2014): “Resources,” “Stress,” “Mental health”]. Furthermore, some studies were based on small sample sizes, which may have led to unreliable models and invalid conclusions (Goh et al., 2010; Gonzalez-Ramirez et al., 2011; Kocalevent et al., 2014). Another drawback was that most of the previous studies analyzed very specific samples such as primiparous women or teachers (Supplementary Table 1).

Therefore, the aim of our study was to test a structural model based on the transactional stress concept by Lazarus and Folkman in a sufficiently large sample of patients with a wide range of health conditions that received inpatient treatment in a psychosomatic clinic. A sample of psychosomatic inpatients seemed to be well suited for the analysis of a stress model as many of the psychosomatic disorders can be associated with elevated stress levels or stressful life events (Cohen, 2000; Nakao, 2010). In contrast to previous studies that tested the Transactional Model using the PSQ (Kocalevent et al., 2007, 2014; Heinen et al., 2017), we focused on the person–environment interaction by modeling personal resources and perceived external stressors as the antecedents, coping as the mediating process, stress response as the immediate effect, and depression as the long-term effect (see Figure 1).

## Materials and Methods

### Sample and Procedure

Data were collected electronically via personal digital assistants (PDAs) as part of the clinical routine assessment at the inpatient clinic of the Department of Psychosomatic Medicine at Charité – Universitätsmedizin Berlin, Germany, between December 2007 and March 2014. The psychosomatic inpatient population encompassed a wide range of health conditions including somatoform disorders, eating disorders, and chronic pain disorders as well as physical conditions associated with mental disorders, such as depression and anxiety. The initial dataset included 2,359 cases. Only those cases with complete datasets (i.e., where patients had answered all instruments) were included in the cross-sectional analyses. Cases with incomplete data on any questionnaire were excluded (*n* = 145) leading to a total of *n* = 2,216 cases included in the study. Sensitivity analyses were carried out to evaluate whether the excluded cases differed from the included cases in their clinical or demographic characteristics. As the data were assessed electronically and as patients were forced to answer each item before the assessment was continued, there were no missing items within each assessed questionnaire. The sample was randomly split into two subsamples to allow model building in the first dataset (sample 1, *n* = 1,129) and confirmatory analyses in the second dataset (sample 2, *n* = 1,087).

### Instruments

In the following paragraphs, we present the items used as indicator variables for the unobserved latent variables as well as additional data assessed in this study.

#### Resources

The latent factor *Resources* was built based on items of two different instruments that capture sense of coherence, self-efficacy and optimism, considering that these constructs have been identified as important protective factors of health (Eriksson and Lindstrom, 2006; Kroninger-Jungaberle and Grevenstein, 2013; Kim et al., 2017).

#### Sense of Coherence

Antonovsky (1979) defined the construct *Sense of Coherence* (SOC) in his salutogenetic model. It consists of three interrelated facets: comprehensibility, manageability, and meaningfulness. Based on Antonovsky’s 29-item Sense of Coherence scale (SOC-29) (Antonovsky, 1983), the Leipzig Short Scale (SOC-L9) was developed, which was used in this study. It consists of nine items and captures one global SOC factor. The SOC-L9 has demonstrated good psychometric properties; the internal consistency (Cronbach’s alpha) found in the development study was 0.87 (Schumacher et al., 2000).

#### Self-Efficacy and Optimism

*Self-efficacy* is a construct that captures the belief in one’s ability to deal with environmental demands (Bandura, 1997; Benight and Bandura, 2004). *Optimism* can be defined as a positive expectation toward one’s future (Scheier and Carver, 1992; Carver and Scheier, 2014). The subscales *self-efficacy* (five items) and *optimism* (two items) of the Self-efficacy, optimism, pessimism short scale (SWOP-K9) were administered in this study. The *self-efficacy* subscale and the *optimism* subscale showed appropriate psychometric properties in the development study. Internal consistencies (Cronbach’s alpha) were 0.86 and 0.78 for self-efficacy and optimism, respectively (Scholler et al., 1999).

#### Perceived Stressors

The latent factor *Perceived Stressors* was created using the five items included in the *demands* subscale of the Perceived Stress Questionnaire (PSQ) (Levenstein et al., 1993; Fliege et al., 2001, 2005). The PSQ was specifically designed for clinical research in psychosomatic patients, as the psychosomatic influences on structural changes of the body are difficult to assess given their subtlety (Levenstein et al., 1993). The original version with 30 items (Levenstein et al., 1993) has been shortened to 20 items. Along with this adaption, the original seven-factor structure was revised resulting in a four-factor solution with the subscales *demands*, *tension*, *worries*, and *joy* (Fliege et al., 2005). The 20-item version has been validated in several studies and has demonstrated sufficient psychometric properties (Fliege et al., 2001, 2005; Kocalevent et al., 2007). The items of the *demands* subscale assess the individual subjective perception of environmental stressors. The items are worded such that they are applicable to respondents in different situations, but still reflect relevant stressors in everyday life (e.g., “I feel under pressure from deadlines.”). The *demands* subscale showed an internal consistency (Cronbach’s alpha) of 0.79 (Fliege et al., 2005).

#### Coping

The latent factor *Coping* was included in the stress model to assess the mediating process between *Resources*, *Perceived Stressors*, and *Stress Response* as proposed by Lazarus and Folkman (1987). Although *Appraisal* is part of the transactional stress model, it was not directly measured in this study. However, Lazarus and Folkman suggested that *Coping* is closely related to the concept of cognitive appraisal. They defined Coping as “the cognitive and behavioral efforts made to master, tolerate, or reduce external and internal demands and conflicts among them” (Folkman and Lazarus, 1980, p. 223). The Brief Coping Questionnaire (Brief COPE, Carver, 1997) was used to operationalize coping in this study. It includes a total of 28 items. Four latent factors *Evasive coping*, *Support seeking*, *Focus on positive*, and *Active coping* were identified for the German version of the Brief COPE by Knoll et al. (2005). *Evasive coping*, *Support seeking*, *Focus on positive*, and *Active coping* demonstrated acceptable internal consistencies (Cronbach’s alpha) of 0.70, 0.76, 0.76, and 0.81, respectively (Knoll et al., 2005).

#### Stress Response

The latent factor *Stress Response* was created by the three dimensions *tension*, *worries*, and *joy* of the Perceived Stress Questionnaire (PSQ, Fliege et al., 2005). The *Stress Response* factor reflects emotional responses (e.g., “You feel tense.”), originally described by Lazarus and Folkman (1987) as an immediate stress reaction. Each subscale of the PSQ consists of five items. Fliege et al. (2005) suggested that the PSQ assesses two distinct stress dimensions, which is in line with previous findings of a two-factorial structure of stress (Lobel and Dunkel-Schetter, 1990). While the *demands* subscale captures the perception of environmental stressors, the *tension, worries*, and *joy* subscales—which are used to operationalize *Stress Response* in our study—measure stress reaction. The *worries* subscale, the *tension* subscale, and the *joy* subscale showed internal consistencies (Cronbach’s alpha) of 0.83, 0.80, and 0.83, respectively (Fliege et al., 2005).

#### Psychological Well-Being

*Psychological well-being* was proposed as the outcome in the original Transactional Model by Lazarus and Folkman (1987). To operationalize *psychological well-being* in this study, we used depressive symptoms as these reflect one major sub-construct of *psychological well-being*. The latent factor *Depression* was modeled with the items of the Patient Health Questionnaire nine-item depression scale (PHQ-9) (Kroenke et al., 2001). The PHQ-9 covers all aspects of depressive symptoms, as proposed in the Diagnostic and Statistical Manual of Mental Disorders, fourth edition (DSM-IV). The PHQ-9 has demonstrated good psychometric properties. The internal consistency (Cronbach’s alpha) was 0.89 in primary care patients (Kroenke et al., 2001).

#### Sociodemographic and Clinical Data

In addition, sociodemographic data (age, gender, education etc.) were assessed. As part of the clinical routine assessment, psychosomatic patients answered the ICD-Symptom Rating (ISR) including 29 items, covering five syndrome scales (Depression, Anxiety, Obsessive-compulsive, Somatoform, Eating disorder) and a supplementary scale (Tritt et al., 2008).

### Statistical Analyses

Descriptive analyses were performed using IBM SPSS Statistics 24. *T*-Tests and Mann–Whitney *U* tests (non-parametric data) were used to compare characteristics between subsamples and in- and excluded cases. Structural equation modeling was carried out using the *lavaan* package (Rosseel, 2012) in R 4.0.3 (R Development Core Team, 2008). As the indicator variables were measured on 4- to 7-point-Likert scales, the weighted least squares means and variance adjusted estimator (WLSMV) was used, as suggested for ordinal data (DiStefano and Morgan, 2014). To allow model identification, the path from the first indicator variable to the latent variable was fixed to 1. Criteria for acceptable model fit were Comparative Fit Index (CFI) and Tucker–Lewis Index (TLI) > 0.9, root mean square error of approximation (RMSEA) < 0.08, and standardized root mean square residual (SRMR) < 0.08 (Browne and Cudeck, 1993; Kline, 2010; Little, 2013).

#### Model Building Analyses (Sample 1, *n* = 1,129)

The initial model was created to closely reflect the transactional stress concept of Lazarus and Folkman (1987). Thus, it included the impact of *Resources* on *Perceived Stressors* (primary appraisal), the influence of *Resources* and *Perceived Stressors* on *Coping* (secondary appraisal), the impact of *Coping* on *Stress Response*, and the effect of *Stress Response* on *Psychological well-being (i.e., Depression)* as shown in Figure 1 (bottom model). As suggested by the literature, *Coping* mediates the pathways from *Perceived Stressors* to *Stress Response* and from *Resources* to *Stress Response*, which in turn predicts *Psychological well-being*. *Resources* were hypothesized to attenuate *Perceived Stressors*, to enable appropriate *Coping* strategies and thereby to weaken the *Stress Response*.

To account for the multidimensional structure of the latent factors *Resources*, *Coping*, and *Stress Response*, second-order factor models were used. Whereas the first-order factors were measured by the items of each dimension (e.g., the first-order factor *Sense of Coherence* was measured by the items SOC01 to SOC09), the second-order factors were measured by the first-order factors (e.g., the second-order factor *Resources* was measured by the first-order factors *Sense of Coherence*, *Self-efficacy*, and *Optimism*).

First, we evaluated the fit of the individual measurement models by estimating first-order and second-order confirmatory factor analysis models. In a second step, the whole stress model was evaluated. To avoid overfitting the model to the specific population used in this study, modification indices were used cautiously. In fact, in this study, modification indices were only used in one case to guide the decision on removing a single item from the model.

#### Confirmatory Analyses (Sample 2, *n* = 1,087)

The final structural model obtained in sample 1 was estimated in sample 2 for confirmatory analyses.

## Results

### Sociodemographic Characteristics

Sociodemographic data of the two subsamples are presented in Table 1. We did not find any significant differences between the subsamples. On average, 80% of the two subsamples exceeded the cut-off score for mild symptom stress, 65% exceeded the cut-off for moderate symptom stress, and 24% exceeded the cut-off for severe symptom stress according to the ICD-10 Symptom Rating (ISR) Total score. Sensitivity analysis comparing in- and excluded cases showed that excluded cases were somewhat older (79 vs. 67 years) and that fewer people within the excluded patients were working full- or part-time (see Supplementary Table 2). It has to be noted, however, that only about 6% of the initial sample were excluded, which probably does not limit the generalizability much.

**TABLE 1:** Characteristics of the two psychosomatic inpatient samples.

	**Sample 1 (*n* = 1,129)**	**Sample 2 (*n* = 1,087)**	**Group difference *p*-value**
*Sociodemographic characteristics*			
Age in years			
M (SD)	44.4 (14.8)	44.8 (15.4)	0.456
Range	17–86	17–87	
Gender (% female)	66.6	66.7	0.964
Nationality (% German)	92.6	91.0	0.179
Employment status (% working)	44.9	40.8	0.171
Highest education (%)			
University entrance diploma	34.9	36.3	0.688
Secondary school certificate	44.6	42.4	
Primary school certificate	15.1	16.9	
Without certificate	3.3	2.4	
Still in school	0.8	1.3	
Special needs school degree	1.4	0.6	
Partnership status (%)			
Single	33.4	34.4	0.496
Married/with partner	46.8	42.9	
Divorced/separated	17.6	17.9	
Widowed	2.2	4.8	
Clinical characteristics			
ISR [M (*SD*)/% above cut-off*]			
Depression	1.86 (1.02)/80.2	1.90 (1.06)/79.3	0.365
Anxiety	1.50 (1.14)/65.4	1.48 (1.13)/65.9	0.678
Obsessive-compulsive	1.06 (1.05)/49.8	1.07 (1.07)/50.9	0.824
Somatoform	1.28 (1.14)/57.0	1.25 (1.11)/56.4	0.531
Eating disorder	0.69 (1.01)/37.9	0.76 (1.09)/40.7	0.117
ISR total score	1.21 (0.66)/80.6	1.22 (0.67)/79.9	0.723

*ISR, ICD-10-Symptom Ranking. ^∗^Cut-off scores for mild symptom stress are 1 for depressive, anxiety, and obsessive-compulsive syndrome scales; 0.75 for somatoform syndrome scale; 0.67 for eating disorder syndrome scale; and 0.6 for the ISR Total score.*

### Model Building in Sample 1 (*n* = 1,129)

The analyses of the measurement models resulted in acceptable model fit for all latent variables apart from *Coping* (Table 2). To further explore reasons for the insufficient model fit of the latent *Coping* factor, we estimated the measurement models of each of the four coping style factors and found—in contrast to Knoll et al. (2005)—unsatisfactory model fits for all latent coping style factors (*Evasive Coping*: CFI = 0.764, TLI = 0.606, RMSEA = 0.246 [0.230–0.263], SRMR = 0.125; *Support Seeking*: CFI = 0.778, TLI = 0.631, RMSEA = 0.389 [0.373–0.405], SRMR = 0.226; *Focus on Positive*: CFI = 0.795, TLI = 0.659, RMSEA = 0.242 [0.226–0.259], SRMR = 0.126) except for *Active Coping* (CFI = 0.979, TLI = 0.937, RMSEA = 0.146 [0.112–0.182], SRMR = 0.038). These results prompted us (1) to investigate the model fit of other (i.e., non-German) factor solutions, and (2) to develop new, sample-specific factor solutions. We evaluated the original factor structure for the Brief COPE by Carver (1997), which included 14 factors (*Active Coping*, *Planning*, *Positive Reframing*, *Acceptance*, *Humor*, *Religion*, *Using Emotional Support*, *Using Instrumental Support*, *Self-Distraction*, *Denial*, *Venting*, *Substance Use*, *Behavioral Disengagement*, *Self-Blame*), a 4-factor solution in a French sample which included 4 factors (*Seeking Social Support*, *Problem Solving*, *Avoidance*, *Positive Thinking*; Baumstarck et al., 2017), a 7-factor structure by Amoyal et al. (2011) (*Active Coping*, *Avoidant Coping*, *Humor*, *Religion*, *Emotional Support*, *Venting*, *Acceptance*), and a 2-factor solution that was found in Australian project managers (Aitken and Crawford, 2007; *Problem-Focused Coping*, *Emotion-Focused Coping*). In addition, 1-factor, 2-factor, 5-factor, and 9-factor structures were obtained in sample 1 using exploratory factor analysis. Unfortunately, none of the factor solutions was appropriate for inclusion in the structural stress model. The factor models either did not converge at all (literature-informed 14-factor and 2-factor solutions, and exploratory 2-factor solution), or demonstrated negative variances indicating problems with the measurement model (literature-informed 4-factor and 5-factor solutions, and exploratory 5-factor and 9-factor solutions). Thus, despite the unsatisfactory model fit of the factor model from Knoll et al. (2005), we used this factor structure to operationalize *Coping*.

**TABLE 2:** Comparison of model fits and factor loadings for measurement models.

**Measurement model**	**Resources (second order)**	**Perceived stressors**	**Coping (second order)**	**Stress response (second order)**	**Psychological well-being (depression)**
CFI	0.947	0.987	0.707	0.953	0.979
TLI	0.937	0.974	0.669	0.944	0.971
RMSEA	0.103 [0.098–0.108]	0.127 [0.106–0.150]	0.135 [0.131–0.138]	0.097 [0.092–0.103]	0.074 [0.064–0.084]
SRMR	0.047	0.036	0.130	0.048	0.044
Standardized factor loadings (SE) of items	0.54 (0.04)– 0.89 (0.02)	0.71 (0.02)– 0.87 (0.02)	0.37 (0.07)– 0.84 (0.03)	0.68 (0.04)– 0.86 (0.03)	0.57 (0.03)– 0.85 (0.03)
Standardized factor loadings (SE) of second-order factors	0.89 (0.03)– 0.93 (0.03)		0.24 (0.03)– 0.92 (0.13)	0.88 (0.04)– 0.91 (0.04)	

*CFI, Comparative Fit Index; TLI, Tucker–Lewis Index; RMSEA, root mean square error of approximation, 90% CI in square brackets; SRMR, standardized root mean square residual.*

**FIGURE 2:**
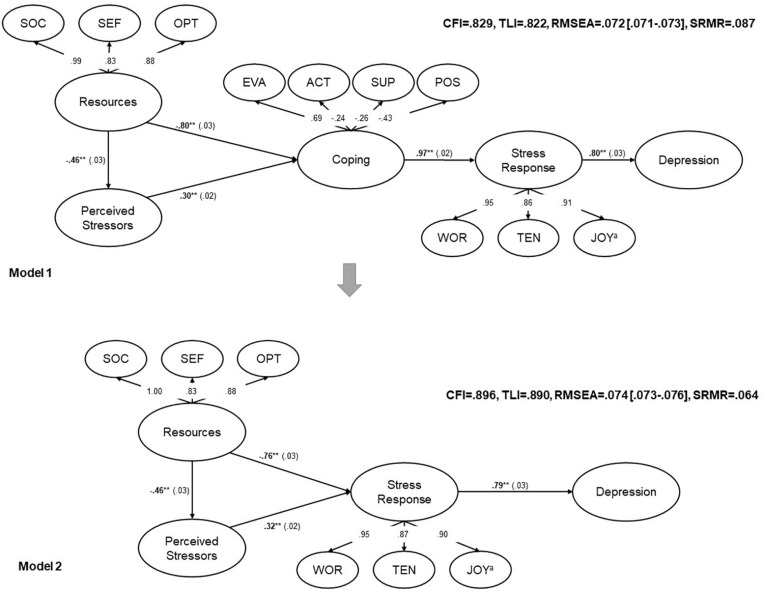
Comparison of the two structural models. Circles: unobserved latent variables, numbers next to the arrows: standardized path coefficients, **p < 0.001, SE in parentheses. ^a^Inverted items on this factor. SOC, *Sense of coherence;* SEF, *Self-efficacy;* OPT, *Optimism;* EVA, *Evasive coping;* ACT, *Active coping;* SUP, *Support seeking;* POS, *Focus on positive;* WOR, *Worries;* TEN, *Tension;* CFI, Comparative Fit Index; TLI, Tucker–Lewis Index; RMSEA, root mean square error of approximation, 90% CI in square brackets; SRMR, standardized root mean square residual.

**FIGURE 3:**
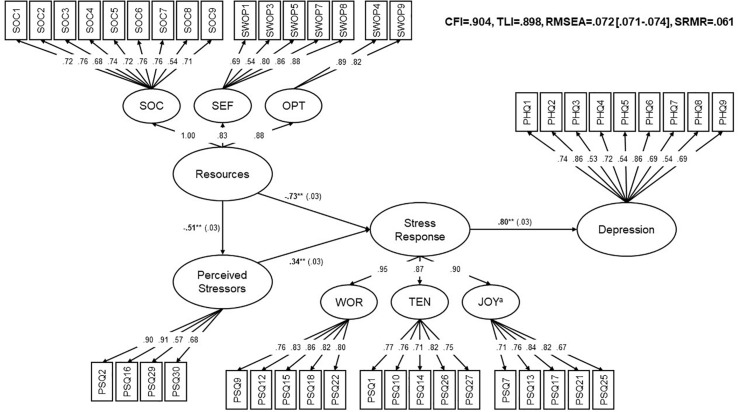
Final Modified Stress Model (model 2 without item PSQ04). Rectangles: observed indicator variables. Circles: unobserved latent variables. For simplification, errors and thresholds were excluded from the figure. Numbers next to the arrows: standardized path coefficients, **p < 0.001, SE in parentheses, ^*a*^Inverted Items on this factor. SOC, *Sense of coherence;* SEF, *Self-efficacy;* OPT, *Optimism;* WOR, *Worries;* TEN, *Tension;* CFI, Comparative Fit Index; TLI, Tucker–Lewis Index; RMSEA, root mean square error of approximation, 90% CI in square brackets; SRMR, standardized root mean square residual.

The estimation of the structural model resulted in insufficient model fit (Figure 2, upper model). In addition, negative residual variances occurred for a small number of coping items indicating that the model was not appropriate for the data (Ullman and Bentler, 2003). The attempt to include modifications as suggested by modification indices (residual covariances between coping items that were loading on the same factors) did not significantly improve the model fit and did not resolve the negative residual variances. Motivated by the good fit of the *Active Coping* factor, we estimated a structural model in which the second-order coping factor (including four coping style factors) was replaced by *Active Coping*. Unfortunately, the model did not converge. Therefore, we decided to exclude the latent *Coping* factor from model 1. The resulting structural model (model 2) is shown at the bottom of Figure 2. The major difference to model 1 is that *Resources* and *Perceived Stressors* predict *Stress Response* directly without being mediated by *Coping*. The resulting fit indices, however, did approach the cut-off of the fit indices but did not meet them. Modification indices showed that the item PSQ04 “You have too many things to do” was suggested to cross-load on several other factors. Therefore, we removed this item from the model (Table 3). The modified model 2 without the PSQ04 item yielded a largely acceptable model fit (CFI = 0.904, TLI = 0.898, RMSEA = 0.072 [0.071–0.074], SRMR = 0.061; Figure 3).

**TABLE 3:** Modification indices (cut-off > 300).

**Variable 1**	**Operator**	**Variable 2**	**Modification index**	**Standardized expected parameter change**
Resources	∼∼	Tension	555.63	1.67
Stressors	∼∼	Tension	440.63	0.52
Joy	=∼	PSQ04	434.99	−0.42
Resources	∼∼	Joy	415.01	−1.66
Stress reaction	=∼	PSQ04	395.80	−0.38
Depression	=∼	PSQ04	390.54	−0.39
Resources	=∼	PSQ04	390.27	0.31
Sense of coherence	=∼	PSQ04	389.97	0.31
optimism	=∼	PSQ04	387.82	0.34
Stressors	∼∼	Joy	378.35	−0.58
Worries	=∼	PSQ04	377.74	−0.38
Tension	=∼	PSQ02	372.94	0.45
Self−efficacy	=∼	PSQ04	368.93	0.32
Worries	=∼	PSQ02	357.09	0.43
Stress reaction	=∼	PSQ02	348.22	0.42
Sense of coherence	=∼	PSQ02	335.86	−0.33
Resources	=∼	PSQ02	335.85	−0.33
Tension	=∼	PSQ16	330.06	0.42
Sense of coherence	=∼	PHQ06	323.30	−0.57
Resources	=∼	PHQ06	323.23	−0.57
Optimism	=∼	PSQ02	300.05	−0.33

*PSQ, Perceived Stress Questionnaire; PHQ, Patient Health Questionnaire; ∼∼ residual covariance; =∼ is measured by.*

*Stress Response* was predicted by *Perceived Stressors* and *Resources* demonstrating an explained variance of 91%. The impact of *Resources* on *Stress Response* was greater than the impact of *Perceived Stressors* on *Stress Response*. *Resources* also influenced *Perceived Stressors*. The impact of *Stress Response* on *Depression* was high which was expressed by an explained variance of 63% (Figure 3).

### Confirmatory Analysis in Sample 2 (*n* = 1,087)

The proposed model was confirmed in the second sample of psychosomatic inpatients, yielding adequate model fit (CFI = 0.932, TLI = 0.928, RMSEA = 0.066 [0.065–0.068], SRMR = 0.052). The standardized path coefficients were very similar to the ones in the exploratory sample (Table 4). To investigate, whether we artificially overfitted the model by deleting the PSQ04 item, we estimated the initial model without this modification in sample 2. This resulted in a slightly lower, but still adequate fit (Table 4).

**TABLE 4:** Comparison of model fits, standardized path coefficients, and explained variance of the final model.

**Structural models**	**Sample 1**		**Sample 2 (confirmatory)**	
	**Model 2**	**Model 2 without item PSQ04**	**Model 2**	**Model 2 without item PSQ04**
CFI	0.896	0.904	0.925	0.932
TLI	0.890	0.898	0.921	0.928
RMSEA [CI]	0.074 [0.073–0.076]	0.072 [0.071–0.074]	0.069 [0.067–0.070]	0.066 [0.065–0.068]
SRMR	0.064	0.061	0.056	0.052
Standardized path coefficients				
• *Resources*→ *Perceived Stressors*	−0.46** (0.03)	−0.51** (0.03)	−0.48** (0.03)	−0.54** (0.03)
• *Resources*→ *Stress Response*	−0.76** (0.03)	−0.73** (0.03)	−0.77** (0.03)	−0.75** (0.03)
• *Perceived Stressors*→ *Stress Response*	0.32** (0.03)	0.34** (0.03)	0.29** (0.02)	0.32** (0.03)
• *Stress Response* → *Depression*	0.79** (0.03)	0.80** (0.03)	0.86** (0.03)	0.86** (0.03)
R^2^ *Perceived Stressors*	0.21	0.26	0.23	0.29
R^2^ *Stress Response*	0.90	0.91	0.90	0.91
R^2^ *Depression*	0.63	0.63	0.73	0.73

**p < 0.001, SE in parantheses, R^2^: explained variance.

## Discussion

This study largely confirmed the theoretical Transactional Model in a slightly modified version by excluding *Coping* in two randomly split psychosomatic subsamples. We were able to show empirically—as hypothesized by Lazarus and Folkman (1987)—that both resources and perceived stressors had an impact on the resulting stress response, which in turn strongly predicted depression. Further, resources had a stronger impact on the following stress response than perceived stressors. Resources did not only attenuate the stress response, but also influenced perceived stressors. These findings confirm the assumption of Lazarus and Folkman (1987) that stress is a highly individual concept resulting from a person–environment transaction. Furthermore, the results underline the importance of strengthening resources in psychotherapy and disease prevention and indicate a high relevance of resilience processes that allow an adaptive stress response in the face of adversity.

*Coping* had to be excluded from the model, as the measurement models for different literature-based and exploratory factor solutions did not fit the data and led to estimation problems in the complex structural stress model. The items of the Brief COPE did not consistently load on the four different factors proposed by Knoll (2002) and Knoll et al. (2005), which resulted in poor model fit. This is in line with previous research that reported different factor structures across different populations which indicates that the population-independent assessment of distinct coping styles remains difficult (Aitken and Crawford, 2007; Amoyal et al., 2011; Baumstarck et al., 2017). As coping is highly contextual and as coping strategies are most often not *per se* adequate or inadequate, they might be actually different between individuals and populations and thus assessment might be challenging. We think that further emphasis should be put on developing better self-report coping instruments or revise existing measures such that they are able to assess coping styles, independent from specific populations. This might be achieved by using modern measurement theory (i.e., item-response theory, IRT) to determine latent uni- or multidimensional coping style factors.

This same approach (i.e., using or even developing measurement models that enable the instrument- and population-independent assessment of latent factors) could potentially improve future analyses of the Transactional Stress Model (and other similar models). Those kinds of analyses would, however, require very large sample sizes to ensure reliable assessment across different populations. Once these measures have been developed, it would probably be far easier to create complex structural models by simply combining different IRT measures such as Stressors, Stress Response, or psychological well-being. Luckily, over the past years, there have been increasing efforts to develop IRT-based measures that are easily applicable in clinical and non-clinical samples, for example by the Patient-Reported Outcomes Measurement Information System (PROMIS) initiative (Cella et al., 2010), and those could be used as a blueprint for the development of specific instruments measuring latent stress, resilience, and coping constructs (Devine et al., 2016; Obbarius et al., 2018).

We empirically confirmed the core piece of the transactional theory, which describes that stress response is induced by a transaction between the person and the environment. This applies even though the pathways for coping and appraisal processes could not be directly analyzed given that *Coping* had to be excluded from the model and given that no direct measures of appraisals were included. Furthermore, it could be shown that depression is highly influenced by the individual response to stress. By modeling the different facets of the stress process, we addressed Lazarus’ claim that as stress is a complex, multivariate process, it has to be measured by a series of instruments that capture the different facets of the stress process (Lazarus, 1990).

### Strengths and Limitations

The strengths of the present study design were that the large sample size allowed to perform consecutive model building and confirmatory analyses in two separate subsamples, and the inclusion of well-established questionnaire items as indicator variables for the latent factors. The Transactional Model from Lazarus and Folkman (1987) attributes significant importance on person–environment interaction and appraisal processes. Therefore, it seems logical that the indicator variables used were based on self-report measures where appraisal processes can be considered as inherent. For example, the Perceived Stress Questionnaire was designed to assess the individually perceived stress with a focus on cognitive appraisal (Levenstein et al., 1993). Another strength was the relevant sample of psychosomatic patients with a broad range of chronic conditions.

The present study has a cross-sectional design. Therefore, hypothesized causal relations must be treated with caution. Longitudinal data are needed to further confirm the Transactional Model. Furthermore, repeated-measurement designs will give more insight into intra-individual variations of the stress process in different encounters. In this way, research could focus more on the process character of stress, as was already suggested by Lazarus as early as 1978 (Lazarus, 1978).

Only patients with complete datasets for all questionnaires were included in the study. *Post hoc* analyses revealed statistically significant differences in gender and employment status between the dropouts and completers. This could be a possible bias of the study. However, compared with the whole sample size (*n* = 2,216), the number of non-completers (*n* = 145) was low.

A cut-off > 0.9 for the model fit indices CFI and TLI is regarded as sufficient fit by some authors (Little, 2013), although other authors propose more strict criteria (e.g., Hu and Bentler (1998) proposed CFI and TLI > 0.95). Yet, there is much debate about the usefulness of cut-off criteria (Heene et al., 2011). Cut-off recommendations should be considered tentative as model results are affected by numerous factors, e.g., sample size, number of indicators, and degree of model misspecification (Marsh et al., 2004). Alternative ways to assess sufficient model fit, for example, in the case of unidimensionality testing for IRT models, have been proposed to overcome the over-rejection of models due to strict cut-off criteria (Reise et al., 2013). Given the use of a robust WLSMV estimator, the consistent results for the model in both subsamples and the absence of indicators for model misspecification, we think that the presented structural model can be regarded as sufficiently proven.

In the past, there was much debate about the potential confounding of stress perception and psychopathology, given that they both rely on self-report measures (Dohrenwend et al., 1984). Yet, the subjectivity of stress measures is an explicit goal given that stress is a product of appraisal in line with Lazarus response to this critique. Research evidence showed that the manipulation of the item wording with the aim to be more objective did not affect the relationship between the stress items and health or well-being to a great extent (Lazarus et al., 1985).

Nevertheless, it would be interesting to include some objective criteria in future studies to evaluate the concordance of objective criteria and the subjective construal of reality.

## Conclusion

The Transactional Model could be confirmed in empirical data of psychosomatic patients, although it was slightly modified by excluding Coping from the model. The main paths hypothesized by Lazarus and Folkman were embedded in the model: Stress response is strongly predicted by individual resources and perceived stressors. The individual stress response in turn highly predicts depression.

## Data Availability Statement

The dataset generated and analyzed for this study can be obtained from the corresponding author upon reasonable request. Requests to access these datasets should be directed to NO, nina.obbarius@charite.de.

## Ethics Statement

The study was reviewed and approved by the Charité’s Ethics Committee. The study was carried out in compliance with the Declaration of Helsinki. Written informed consent for participation was not required for this study in accordance with the national legislation and the institutional requirements.

## Author Contributions

NO and MR planned the study. NO was in charge of the data analyses and drafted the initial version of the article. FF, GL, AO, and MR supported statistical analyses and interpretation. NO and AO drafted the first version of the revised manuscript. All authors discussed each section of the article, and commented on the article, and agreed to be accountable for the content of the work.

## Conflict of Interest

The authors declare that the research was conducted in the absence of any commercial or financial relationships that could be construed as a potential conflict of interest.
