# Supplementary material for: A Modified Version of the Transactional Stress Concept According to Lazarus and Folkman Was Confirmed in a Psychosomatic Inpatient Sample
Source: Front Psychol. 2021 Mar 5;12:584333. doi: 10.3389/fpsyg.2021.584333 (PMC7973375; doi:10.3389/fpsyg.2021.584333)
Supplement: Supplementary file 1 [file Table_1.docx]

**Supplemental Table 1: Summary of studies that aimed to confirm the Transactional Stress Model empirically**

| **Authors and year** | **Study population** | **Sample size** | **Study design** | **Constructs included in the model** | **Statistical technique** | **Main findings** | **Motivation for our study** |
| --- | --- | --- | --- | --- | --- | --- | --- |
| Quine and Pahl 1991 | Mothers caring for a child with learning disabilities | 166 | cross-sectional | Characteristics of the child as stressors,  characteristics of the family as coping resources,  Coping strategies,  Physical and psychological symptoms associated with emotional distress | hierarchical regression analysis | The transactional stress model was supported.  Four of the five coping resources were found to be significant contributors in a hierarchical regression analysis of stress scores, contributing additional variance beyond that of behavioral and other child characteristics. 55% of the variance of stress scores were explained | Specific sample  No SEM |
| Jerusalem 1993 | East German Migrants | 302 | longitudinal | employment status and quality of residential environment  were taken as antecedents to predict appraisals of distress, emotional coping tendencies and  physical health complaints observed 9 months later | structural equation modeling | A considerable predictive power of the theoretical model could be demonstrated. Personal resources and environmental constraints turned out to be strong predictors of stress appraisals, which in  turn had a significant impact on coping and health. Stress appraisals were also found to be the decisive mediator of the antecedents’ effects on these latter variables, as predicted by theory.  Compared to a simpler cause-effect model, the  theoretical assumption of a complex transactional structure yields a distinctly improved representation  of empirical relationships. | Small number of indicators for latent variables  Specific Sample |
| Honey et al. 2003 | primiparous women | T1: 306 (final trimester)  T2: 223 (follow-up 6 weeks after childbirth) | longitudinal | T1:  Depression vulnerability,  Social support,  Appraisal,  Coping style  T2:  Concurrent levels of social support,  Stress,  Appraisal | hierarchical multiple regression | The main premises of the proposed model were upheld. Depression vulnerability and negative antenatal appraisal processes appeared to exert a negative influence on women’s coping behavior and subsequent postnatal appraisals of childcare stress. In turn, these negative appraisal and coping processes and women’s perceptions of poor postnatal support, appeared to exert a negative impact on maternal mood, resulting in low postnatal mood in women vulnerable to depression. | Very Specific Sample/Setting  Lack of diagnostic criteria to assess the incidence of PND  No SEM |
| Bouchard, Guillemette, and Landry-Léger 2004 | French-Canadian university students | T1: 233  T2: 197  (10 weeks later) | longitudinal | Dispositional coping,  Situational coping,  Personality traits,  Cognitive appraisals,  Psychological distress | hierarchical multiple regression analyses | Situational and dispositional coping were related but not redundant constructs, each being determined by its own set of predictors. | Specific Sample  No SEM  Focus on analysis of the relationships between  dispositional and situational coping, by examining their determinants and respective role in  psychological distress |
| Kocalevent et al. 2007 | German  general population | 2552 | cross-sectional | Resources,  Stress perception, Health (Quality of life) | structural equation modeling | A transactional model fitted the data. Personality aspects and resources contributed to the total perceived stress. Yet the physical aspects of quality of life received a comparatively low weighting. | Reduces complexity of the transactional stress model to 3 latent constructs  Subdomains operationalized as observed variables (domain scores) rather than latent variables  Subscale Joy of the Perceived Stress Questionnaire operationalized as a personal resource and not a stress reaction or perceived stress |
| Heinen et al. 2007 | Medical students | 321 | cross-sectional | Personal resources including resilient coping,  Perceived stress, Emotional distress | structural equation modeling | Structural equation analysis revealed a satisfactory fit between empirical data and the proposed stress model  indicating that personal resources modulated perceived stress, which in turn had an impact on emotional distress. | Reduces complexity of the transactional stress model to 3 latent constructs  Resilient coping operationalized as a personal resource and not a mediator  Subscale Joy of the Perceived Stress Questionnaire operationalized as a personal resource and not a stress reaction or perceived stress |
| Laugaa et al. 2008 | French Teachers | T1: 410  T2: 259 (follow-up) | longitudinal | Self-efficacy, Professional stressors,  Perceived social support,  Perceived stress,  Coping strategies,  Burnout | structural equation modeling | The whole constrained and tested model had an adequate fit. The principal independent variables of the model (self-efficacy, professional stressors, perceived social support, perceived stress, and coping strategies) explained 40% of the variance of burnout. | Very Specific Sample |
| Goh et al. 2010 | Australian participants with full time employment | 129 | longitudinal | Primary and secondary stress appraisal,  Coping,  Occupational stress | structural equation modeling | The modified model revealed that in addition to the basic four paths found in Lazarus and Folkman’s transactional model (from primary appraisal to secondary appraisal, from secondary appraisal to stress, and from stress to coping), there was a direct link between primary appraisal and stress level time one and between stress level time one and stress level time two. | Small sample size |
| Gonzalez-Ramirez et al. 2011 | Fibromyalgia patients | 165 | cross-sectional, uncontrolled study | Personal and social resources (self-esteem, self-efficacy, social support),  Perceived stress level,  Symptoms and Impact of Fibromyalgia | structural equation modeling | The model confirmed that some personal and social resources (self-esteem, self-efficacy, social support) perform as predictors of perceived stress level explaining 53% of its variance. This perceived stress was predictor of a latent variable indicated by symptoms and Fibromyalgia impact, and it explained 31% of its variance. | Small sample size |
| Kocalevent et al. 2013 | German  general  population | 2552 | cross-sectional | Resources (self-efficacy, optimism),  Chronic stress, Fatigue | structural equation modeling | Resources had an influence on chronic stress and also a direct influence on fatigue, the influence of chronic stress on fatigue was diminished by taking this direct influence into account. | Reduces complexity of the transactional stress model to 3 latent constructs |
| Hulbert-Williams et al. 2013 | recently diagnosed cancer patients | 160  (3- and 6-Months follow-up) | longitudinal | Appraisal components,  Core-relational themes,  Emotions | Spearman’s correlation tests and multivariate regression modelling | Although data supported the generic structure of the Transactional Model, they questioned the model specifics.  Both primary and secondary appraisals, and core-relational themes were important variables in explaining variance in emotional outcome. Cross-sectional multivariate testing of the ability of cognitions to explain variance in emotion was largely theory inconsistent. Equally high levels of variance could be explained using entirely different cognitive appraisals than those hypothesized. | Specific sample  No SEM  Focus on specific hypotheses about which particular stress appraisals would determine which emotional response |
| Kocalevent et al. 2014 | patients consecutively admitted for adjustment disorders | 108 | longitudinal | Resources,  Stress,  Mental health | structural equation modeling | The transactional stress model did not fit the data. | Reduces complexity of the transactional stress model to 3 latent constructs  Small sample size  Subscale Joy of the Perceived Stress Questionnaire operationalized as a personal resource and not a stress reaction or perceived stress |
| Hulbert-Williams et al. 2013 | recently diagnosed cancer patients | 160  (3- and 6-Months follow-up) | longitudinal | Appraisal components,  Core-relational themes,  Emotions | Spearman’s correlation tests and multivariate regression modelling | Although data supported the generic structure of the Transactional Model, they questioned the model specifics.  Both primary and secondary appraisals, and core-relational themes were important variables in explaining variance in emotional outcome. Cross-sectional multivariate testing of the ability of cognitions to explain variance in emotion was largely theory inconsistent. Equally high levels of variance could be explained using entirely different cognitive appraisals than those hypothesized. | Specific sample  No SEM  Focus on specific hypotheses about which particular stress appraisals would determine which emotional response |
