# Supplementary material for: A Modified Version of the Transactional Stress Concept According to Lazarus and Folkman Was Confirmed in a Psychosomatic Inpatient Sample
Source: Front Psychol. 2021 Mar 5;12:584333. doi: 10.3389/fpsyg.2021.584333 (PMC7973375; doi:10.3389/fpsyg.2021.584333)
Supplement: Supplementary file 2 [file Table_2.docx]

**Supplemental Table 2.** Characteristics of the included and excluded cases

|  | Included (n=2,216) | Excluded (n=145) | Group difference  *p*-value |
| --- | --- | --- | --- |
| ***Socio-demographic characteristics***  Age in years  M (SD) | 44.6 (15.1) | 46.15 (15.8) | 0.308 |
| Gender (% Female) | 66.6 | 78.6 | **<0.001** |
| Nationality (% German) | 91.7 | 84.8 | 0.103 |
| Employment status (% working) | 42.9 | 34.5 | **0.047** |
| Highest education (%)  University entrance diploma  Secondary school certificate  Primary school certificate  Without certificate  Still in school  Special needs school degree | 35.6  43.5  16.0  2.8  1.0  1.0 | 33.1  43.4  8.3  7.6  2.8  1.4 | 0.594 |
| Partnership status (%)  Single  Married/with partner  Divorced/separated  Widowed | 33.9  44.8  17.8  3.5 | 29.0  46.9  15.2  5.5 | 0.436 |
| ***Clinical characteristics***  ISR (M (SD) / % above cut-off*)  Depression  Anxiety  Obsessive-compulsive  Somatoform  Eating disorder  ISR Total score | 1.88 (1.04)  1.49 (1.14)  1.07 (1.06)  1.26 (1.13)  0.73 (1.05)  1.22 (0.66) | 1.85 (1.01)  1.59 (1.14)  1.13 (1.06)  1.24 (1.08)  0.68 (0.97)  1.23 (0.67) | 0.425  0.811  0.786  0.340  0.506  0.903 |

*Abbreviations*: SD, standard deviation; ISR, ICD-10-Symptom Ranking. **Bold** p-value<0.05
